# Determinants of early initiation of breastfeeding in Papua New Guinea: a population-based study using the 2016-2018 demographic and health survey data

**DOI:** 10.1186/s13690-020-00506-y

**Published:** 2020-11-23

**Authors:** Abdul-Aziz Seidu, Bright Opoku Ahinkorah, Ebenezer Agbaglo, Louis Kobina Dadzie, Justice Kanor Tetteh, Edward Kwabena Ameyaw, Tarif Salihu, Sanni Yaya

**Affiliations:** 1 Department of Population and Health, University of Cape Coast, Cape Coast, Ghana; 2 College of Public Health, Medical and Veterinary Sciences, James Cook University, Townsville, Queensland Australia; 3 School of Public Health, Faculty of Health, University of Technology Sydney, Sydney, Australia; 4 Department of English, University of Cape Coast, Cape Coast, Ghana; 5 School of International Development and Global Studies, University of Ottawa, Ottawa, Canada; 6 The George Institute for Global Health, The University of Oxford, Oxford, UK

**Keywords:** Breastfeeding, Early initiation of breastfeeding, Newborn health, Papua New Guinea, Public health, Global health

## Abstract

**Background:**

Initiation of breastfeeding after birth comes with a wide range of benefits to the child. For example, it provides the child with all essential nutrients needed for survival within the first six months of birth. This study sought to determine the prevalence and factors associated with early initiation of breastfeeding (EIB) in Papua New Guinea.

**Methods:**

We utilized the Demographic and Health Survey data of 3198 childbearing women in Papua New Guinea. We employed descriptive and binary logistic regression analyses. We presented the results as Crude Odds Ratios (COR) and Adjusted Odds Ratios (AOR), with 95% confidence intervals (CI) signifying level of precision. Level of statistical significance was set at *p* < 0.05.

**Results:**

Women aged 20–29 [AOR = 1.583, CI = 1.147–2.185] and those aged 30+ [AOR = 1.631, CI = 1.140–2.335] had higher odds of EIB, compared to those aged 15–19. Women from the Islands region had lower odds [AOR = 0.690, CI = 0.565–0.842] of EIB, compared to those in Southern region. Women who delivered through caesarean section had lower odds of EIB, compared to those who delivered via vaginal delivery [AOR = 0.286, CI = 0.182–0.451]. Relatedly, women who delivered in hospitals had lower odds of EIB [AOR = 0.752, CI = 0.624–0.905], compared to those who delivered at home. Women who practiced skin-to-skin contact with the baby [AOR = 1.640, CI = 1.385–1.942] had higher odds of EIB, compared to those who did not. Women who read newspaper or magazine at least once a week had lower odds of EIB [AOR = 0.781, CI = 0.619–0.986], compared to those who did not read newspaper at all.

**Conclusion:**

The prevalence of EIB in Papua New Guinea was relatively high (60%). The factors associated with EIB are age of the women, region of residence, mode of delivery, place of delivery, practice of skin-to-skin contact with the baby, and exposure to mass media (newspaper). To increase EIB in Papua New Guinea, these factors ought to be considered in the implementation of policies and measures to strengthen existing policies. Health providers should educate mothers on the importance of EIB.

## Background

Early initiation of breastfeeding (EIB) is noted to come with a wide range of benefits to the child [1]. In the first place, it provides the child with all essential nutrients needed for survival within the first six months of birth [2]. Research suggests that EIB supplies the child with immunoglobin, which boosts the child’s immune system and promotes the growth and development of the child [3]. Besides, globally, breastfeeding helps to save the lives of about 22% of children who die neonatally of diseases such as diarrhoea, meningitis [4], and pneumonia [5, 6]. It has also been noted that EIB creates some form of bonding between the mother and child, which is necessary for the child’s development [7]. On the other hand, breastfeeding, when delayed, leads to high prevalence of infant mortality [8, 9].

In view of the benefits of early breastfeeding, the World Health Organization (WHO) recommends that it should be initiated within the first hour of birth and this is termed as Early Initiation of Breastfeeding (EIB) [10]. EIB has seen much support from national and international bodies [11]. Despite this, over the years, there have not been any significant improvement in EIB globally [12]. Globally, less than half of newborns are breastfed within the first hour, and even in 2017, only 22% of all newborns were breastfed in the first hour of birth. In East Asia and the Pacific, only 32% of newborns meet the WHO’s recommendation of EIB [13]. In Papua New Guinea, the attempt by the government to protect breastfeeding through the passing of the Baby Food Supplies (Control) Act 1977 has yielded little results [8]. While nationally representative information on determinants of EIB is scarce in Papua New Guinea, studies by Kuzma [8] and Goris et al. [14] indicate a prevalence of 69% and 36.1% in rural and isolated communities in Gulf Province and Madang Province, respectively.

Studies abound on child breastfeeding practices, with focus on Bangladesh [15, 16], Ethiopia [17–20], Nepal [21], Niger [22], Nigeria [23], Saudi Arabia [24, 25], Tanzania [26], Uganda [27], and Zimbabwe [28, 29]. Such research have principally focused on trends of timely initiation of breastfeeding [30], predictors of delayed initiation of breastfeeding [25, 27] and determinants of EIB [17–22, 26]. Age, religion [30], parity [28], mother’s educational status [21], and geographical region of residence [16, 19] have been reported to be associated with EIB. In Papua New Guinea, generally, while there are few studies on infant feeding practices [8], such studies did not consider its associated factors. Papua New Guinea records an overwhelmingly high percentage of child deaths in the Pacific. Also, this lack of research on the subject in the context of Papua New Guinea means a lack of empirical basis for policy interventions and programs aimed at improving EIB which can reduce child deaths. The present study, therefore, aimed at investigating the prevalence and determinants of EIB in Papua New Guinea. Findings from the study will provide target areas for interventions and programs aimed at improving EIB in Papua New Guinea and go a long way to reduce child deaths in the country.

## Methods

### Data source and sample

The data used for this study forms part of the 2016–2018 Papua New Guinea Demographic and Health Survey (PDHS), which was collected from October 2016 to December 2018. A two-stage stratified sampling procedure was used to sample census units (CUs) from each stratum. Stage one involved the selection of 800 CUs. This was done through probability proportional to CU size [31]. The second stage saw the systematic selection of 24 households from each cluster through probability sampling, and this yielded a total of 19,200 households. For this study, we focused on women who had given birth 2 years prior to the survey, and such women numbered 3198, all of which had complete information on the variables the present study was interested in [31]. Details of the methodology, pretesting, training of field workers, the sampling design, and selection are available in the PDHS final report, accessible via the following link: https://dhsprogram.com/publications/publication-fr364-dhs-final-reports.cfm.

### Study variables

#### Outcome variable

The outcome variable was EIB. In the PDHS woman’s questionnaire, mothers were asked “How long after birth did you first put (NAME) to the breast?” Responses were recorded in number of hours or days [19, 32]. Our outcome variable “EIB” was defined as initiation of breastfeeding within 1 h of birth and was expressed as a dichotomous variable with category 1 for initiation of breastfeeding within 1 h (early) and category 0 for initiation of breastfeeding after 1 h (late). This indicator was self-reported by mothers.

#### Independent variables

Fifteen independent variables that were theoretically and empirically related to EIB were considered in this study [8, 19, 30, 32, 33]. These are mother’s age at childbirth, mother’s education, mother’s religion, birth order of child, number of ANC visits, region, residence, wealth, mode of delivery, place of delivery, child size at birth, skin-to-skin contact, frequency of reading newspaper/magazine, frequency of listening to radio, and frequency of watching television. Some of these variables were recoded for meaningful and easy interpretation of results. They include mother’s age at childbirth (less than 20, 20–29, 30-49), mother’s education (no education, primary, secondary or higher), mother’s religion (orthodox, protestants, other), birth order of child (1, 2–3, 4 or more), and number of ANC visits (0, 1–3, 4 or more) (see Table 1).

**Table 1:** Sociodemographic characteristics of women and prevalence of early initiation of breastfeeding in Papua New Guinea, 2016–2018

Variable	Sample *N* = 3198		EIB	EIB
	*N*	%	*n*	%
**Mother's age at childbirth**				
Less than 20	193	6.04	94	48.7
20–29	1725	53.94	1042	60.41
30-49	1280	40.03	778	60.78
**Mother's education**				
No education	635	19.86	385	60.63
Primary	1628	50.91	978	60.07
Secondary or higher	935	29.24	551	58.98
**Mother’s religion**				
Orthodox	1045	32.68	625	59.81
Protestants	1484	46.4	912	61.46
Other	669	20.92	377	56.35
**Wealth status**				
Poorest	538	16.82	329	61.15
Poorer	520	16.26	317	60.96
Middle	628	19.64	390	62.1
Richer	780	24.39	452	57.95
Richest	732	22.89	426	58.2
**Place of Residence**				
Urban	722	2.58	410	56.79
Rural	2476	77.42	1504	60.74
**Region**				
Southern region	961	30.05	607	63.16
Highlands region	794	24.83	472	59.45
Momase region	657	20.54	402	61.19
islands region	786	24.58	433	55.09
**Birth order of child**				
1	759	23.73	420	55.34
2–3	1308	40.9	804	61.47
4+	1131	35.37	690	61.01
**Number of ANC visits**				
No ANC	759	23.73	474	62.45
1–3	776	24.27	466	60.05
4+	1663	52	974	58.57
**Mode of delivery**				
Vaginal delivery	3096	96.81	1887	60.95
Caesarean section	102	3.19	27	26.47
**Place of delivery**				
Health facility	1992	62.29	1157	58.08
Home	1206	37.71	757	62.77
**Child size at birth**				
Small	737	23.05	426	57.8
Average	1208	37.77	742	61.42
Large	1253	39.18	746	59.54
**Skin-to-skin contact**				
No	1542	48.22	862	55.9
Yes	1656	51.78	1052	63.53
**Frequency of reading newspaper/magazine**				
Not at all	2084	65.17	1274	61.13
Less than once a week	622	19.45	377	60.61
At least once a week	492	15.38	263	53.46
**Frequency of listening to radio**				
Not at all	2072	64.79	1258	60.71
Less than once a week	582	18.2	344	59.11
at least once a week	544	17.01	312	57.35
**Frequency of watching Television**				
Not at all	2480	77.55	1494	60.24
Less than once a week	305	9.54	194	63.61
At least once a week	413	12.91	226	54.72

Source: PDHS (2016–2018)

### Statistical analysis

Both descriptive and inferential analyses were conducted. We used descriptive statistics to describe the study sample and the prevalence of EIB across all the independent variables. After that, we built two logistic regression models and reported the results as crude and adjusted odds ratios (see Table 2). The model fitness specification was done with the Hosmer-Lemeshow test while multicollinearity was checked using the variance inflation factor (VIF), which showed no evidence of multicollinearity. We applied sample weight to take care of under and over sampling. We used the svy command to take care of the multi-stage sampling approach of the survey. We used STATA version 14.2 for MacOS to carry out the analyses, and statistical significance was set at *p* < 0.05.

**Table 2:** Crude (unadjusted) and adjusted odds ratios of determinants of early initiation of breastfeeding in Papua New Guinea, 2016–2018

Variables	EIB	
	COR[95%CI]	AOR[95%CI]
**Mother's age at childbirth**		
Less than 20	**Ref**	**Ref**
20–29	1.607**[1.192,2.165]	1.583**[1.147,2.185]
30+	1.632**[1.205,2.212]	1.631**[1.140,2.335]
**Mother's education**		
No education	**Ref**	
Primary	0.977 [0.810,1.179]	–
Secondary or higher	0.932 [0.758,1.145]	–
**Mother’s religion**		
Orthodox	**Ref**	
Protestants	1.071 [0.911,1.260]	–
Other	0.868 [0.713,1.056]	–
**Wealth**		
Poorest	**Ref**	
Poorer	0.992 [0.775,1.270]	–
Middle	1.041 [0.821,1.319]	–
Richer	0.875 [0.700,1.095]	–
Richest	0.884 [0.705,1.110]	–
**Residence**		
Urban	**Ref**	
Rural	1.177 [0.995,1.393]	
**Region**		
Southern region	**Ref**	**Ref**
Highlands region	0.855 [0.705,1.037]	0.87 [0.713,1.060]
Momase region	0.919 [0.749,1.128]	0.869 [0.703,1.075]
Islands region	0.715***[0.590,0.867]	0.690***[0.565,0.842]
**Birth order of child**		
1	**Ref**	**Ref**
2–3	1.288** [1.074,1.544]	1.146 [0.940,1.398]
4+	1.263* [1.048,1.522]	1.084 [0.853,1.378]
**Number of ANC visits**		
No ANC	**Ref**	Ref
1–3	0.904 [0.736,1.110]	–
4+	0.85 [0.713,1.014]	–
**Mode of delivery**		
Vaginal delivery	**Ref**	**Ref**
Caesarean section	0.231***[0.148,0.360]	0.286***[0.182,0.451]
**Place of delivery**		
Health facility	0.822** [0.710,0.952]	0.752** [0.624,0.905]
Home	**Ref**	**Ref**
**Child size at birth**		
Small	**Ref**	Ref
Average	1.162 [0.965,1.401]	–
Large	1.074 [0.893,1.292]	–
**Skin-to-skin contact**		
No	**Ref**	**Ref**
Yes	1.374***[1.192,1.583]	1.640***[1.385,1.942]
**Frequency of reading newspaper/magazine**		
Not at all	**Ref**	**Ref**
Less than once a week	0.978 [0.814,1.175]	0.994 [0.812,1.218]
At least once a week	0.730** [0.599,0.890]	0.781*[0.619,0.986]
**Frequency of watching TV**		
Not at all	**Ref**	**Ref**
Less than once a week	1.153 [0.901,1.476]	1.141 [0.875,1.488]
At least once a week	0.798*[0.647,0.984]	0.885 [0.692,1.131]
**Frequency of listening to radio**		
Not at all	**Ref**	
Less than once a week	0.935 [0.775,1.128]	–
At least once a week	0.87 [0.719,1.054]	–
N		3198
pseudo R^2^		0.028

* *p* < 0.05, ** *p* < 0.01, *** *p* < 0.001, *Ref* reference, *COR* Crude Odds Ratio, *AOR* Adjusted Odds Ratio

Exponentiated coefficients; 95% confidence intervals in brackets

Source: PDHS (2016–2018)

## Results

### Prevalence of EIB

**Fig. 1:**
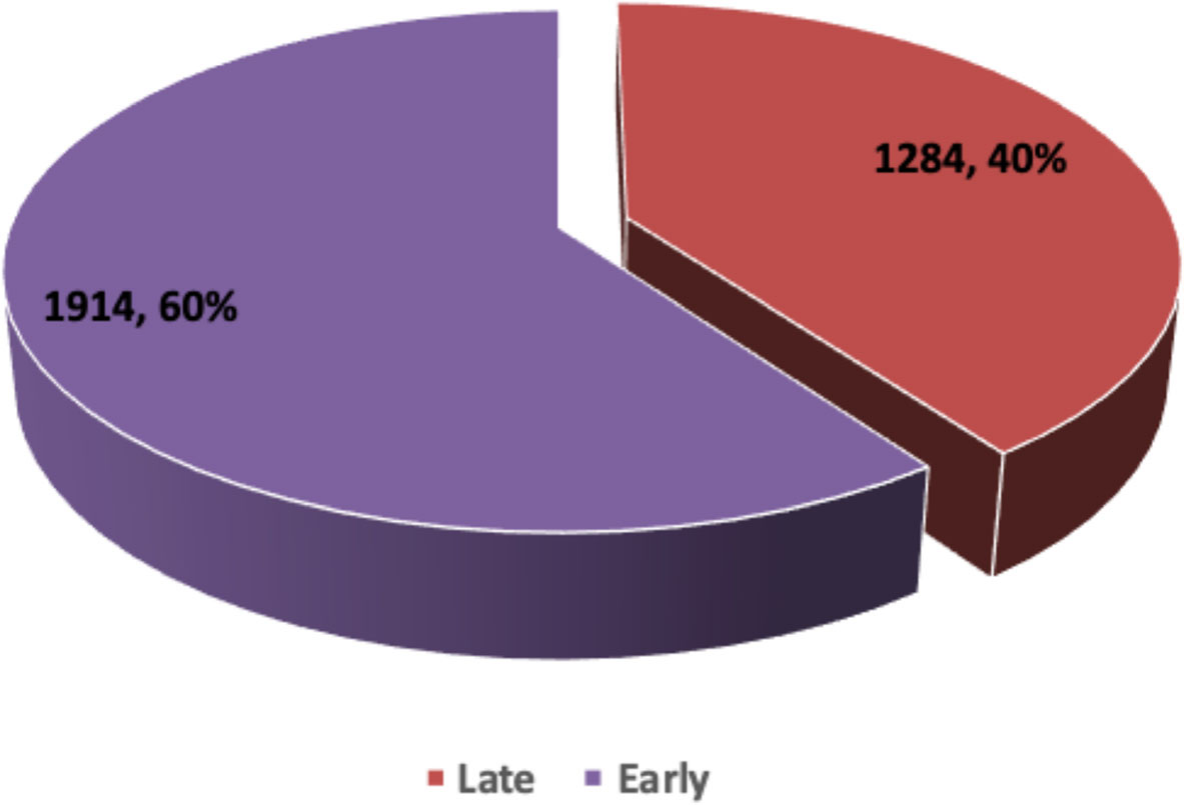
Prevalence of Early Initiation of Breastfeeding in Papua New Guinea, 2016–2018. Source: PDHS (2016–2018)

Figure 1 shows the prevalence of EIB among women in Papua New Guinea. It was found that 60% of mothers breastfed their children within the first 1 h after delivery.

### Sample characteristics and EIB behaviours among women in Papua New Guinea

Table 1 shows the background characteristics of the women. We found that 53.9% were aged 25–29. Approximately 50.9% had primary level of education and 46.0% were Protestants. With wealth status, 24.9% were in the richer wealth category. Also, 7.4% were in rural areas. Similarly, 20.1% were in the Southern region. With EIB, generally, 60.0% of women in Papua New Guinea initiated breastfeeding within the first hour after delivery. Specifically, 60.4% of  women aged 20–29, 60.6% of those with no education, 61.5% of Protestants, 62% of those in the middle wealth quintile, 60.7% of those in rural areas, 63.2% of those in southern region, and 63.6% of those who watched television less than once a week had the highest proportions of EIB (Table 1).

### Unadjusted and adjusted odds ratios of determinants of EIB in Papua New Guinea

Women aged 20–29 [AOR = 1.583, CI = 1.147–2.185] and those aged 30+ [AOR = 1.631, CI = 1.140–2.335] had higher odds of EIB, compared to those aged 15–19. In terms of region of residence, women from the Islands region had lower odds [AOR = 0.690, CI = 0.565–0.842] of EIB, compared to those in Southern region. In relation to mode of delivery, women who delivered through caesarean section (CS) had lower odds of EIB, compared to those who delivered via normal delivery [AOR = 0.286, CI = 0.182–0.451]. Relatedly, women who delivered in hospitals had lower odds of EIB [AOR = 0.752, CI = 0.624–0.905], compared to those who delivered at home. Women who practiced skin-to-skin contact with the baby [AOR = 1.640, CI = 1.385–1.942] had higher odds of EIB, compared to those who did not. Women who read newspaper or magazine at least once a week had lower odds of EIB [AOR = 0.781, CI = 0.619–0.986], compared to those who do not read newspaper at all (Table 2).

## Discussion

The Sustainable Development Goal (SDG) target 3.2 is a wakeup call for all countries to work in ending preventable deaths of newborns and reduce neonatal mortality to, at least, as low as 12 per 1000 live births and under-5 mortality to, at least, as low as 25 per 1000 live births by 2030 [34]. EIB has been identified by WHO as key to achieving this goal due to the associated health benefits such as increased ability to defend against infections, reduced risk of diarrhea, and increased survival rate of children [32]. It has, therefore, become important for health systems globally to understand some key factors that affect EIB. In the present study, we investigated the determinants of EIB in Papua New Guinea, given the high child mortality rate in the country [35], with the overall aim of producing findings that can serve as basis for policy interventions aimed at preventing child deaths in the country. This section discusses key findings of the study.

The results revealed that 60% of mothers in Papua New Guinea breastfed their children within the first hour after delivery, which is similar to what was reported in Bangladesh (51%) [16] and Zimbabwe (58.3%) [30]. This result is, however, lower than the prevalence in many other low- and middle-income countries. For example, 74.3–83.7% was recorded in Ethiopia [17, 19, 36, 37], 76.9% in Malawi [38], 77.74% in Mozambique, 81.51% in Rwanda [33], and 68.6% in Uganda [39]. On the other hand, the prevalence is higher than what was reported in India (36.4%) [40] and 24% in Pakistan [41]. The possible reason for the differences in study findings might be differences in geographical locations/settings, the time differences the studies were conducted and socio-cultural practices as well as differences in the rate of caesarean deliveries [18, 23]. The relatively low EIB recorded in the present study could be explained in the context of some socio-cultural beliefs in the country. For instance, mothers in Papua New Guinea believe that colostrum is dirty and unclean and thus can harm their babies [8]. In the light of this finding, it is, therefore, imperative for the government to implement policies to educate women on the importance of colostrum to their babies. Such policies can help rectify this misconception held by the mothers and boost early initiation of breastfeeding in the country. Thus, the development of the UNICEF-Papua New Guinea Country Program 2018–2022, which aims to build capacity of the most deprived provinces in the country to improve nutrition-focused interventions [42], is in the right direction.

The results showed that mothers who were more than 20 years of age had higher odds of EIB, compared to those aged 19 and below who would probably be having their 1st birth. Duodu et al. [32] have reported similar findings from their study in Ghana, which suggests that infants who were not firstborns had higher likelihood of achieving EIB. This significant finding from the study is also in support of findings in Sri Lanka [43], India [44], Pakistan [45], Namibia [46], and Ethiopia [19]. The reason could be that mothers who are more than 20 years old at birth could likely be having their 2nd birth and might have already been exposed to benefits of EIB through postnatal care for their first child and antenatal care. As such, they will be much conscious and knowledgeable about reaping the benefits associated with EIB. In some situations, younger mothers and those going through delivery for the first time often become tired, exhausted, and may need time to recover from delivery complications with the potential of missing EIB. Evidently, an earlier study in Papua New Guinea reported good knowledge and positive attitudes to breastfeeding among older women [47]. It is therefore important for health workers to educate mothers on the importance of EIB especially younger mothers during ANC and PNC.

There was also a significantly strong association between mode of delivery, place of delivery, and EIB in Papua New Guinea. The mothers who delivered through C-Section had lower odds of EIB, compared to those who delivered via normal vaginal delivery. Relatedly, women who delivered in a hospital had lower odds of EIB, compared to those who delivered at home. This finding is consistent with many findings in low- and middle-income countries such as Ghana [32], Kenya [48], Nepal [49], Zimbabwe [50], and Nigeria [23]. The reasons could be that, after a mother goes through CS delivery, she needs time to gain consciousness from the anesthesia and any complications resulting from the process, thereby putting them at risk of losing EIB [51]. For example, a study by Scott el al [52]. has revealed that delivery by CS-section has physiologic effect on lactogenesis, which results in delays in lactation, which prevents EIB. Some of the barriers posed by CS could be lengthy post-delivery hospital stays, prolonged mother-child separation, delayed skin-to-skin contact, and maternal endocrinological diseases [23]. Also, all CS are conducted in the health facility and this could explain why it is reported in the study that mothers delivering in the health facility had lower prospects of EIB. In Bangladesh, for instance, it is reported that C-Section, possibly, is another reason for lower EIB among mothers who delivered at health facilities than at home, as over 60% of childbirths at health facilities are conducted by C-section [53]. However, this finding is in sharp contrast with what has been reported by Adhikari et al. [54] in Nepal, who found that health facility delivery had a positive influence on EIB. Specifically, Adhikari et al. [54] noted that children born in health facilities were more likely to be breastfed within one hour, compared to those born at home. Further studies could explore health facility delivery, caesarean section and EIB.

We also observed that women who practiced skin-to-skin contact with the baby had higher odds of EIB, compared to those who did not. This finding is in support of findings from a quasi-experimental study in Iraq by Safari et al. [55] which reported that mothers who had skin-to-skin contact with their newborns were more likely to practice EIB. Furthermore, studies conducted in USA by Moore and Anderson [56], in Iran by Khadivzadeh and Karimi [57], and in Pakistan by Mahmood et al. [58] have all reported affirmatively that mothers are more likely to practice EIB when they have skin-to-skin contact with their newborns. The reason could be that early skin-to-skin contact results in higher breastfeeding self-efficacy in mothers and gives mothers confidence in their ability to breastfeed their newborns [58, 59].

Moreover, the study revealed an inverse relationship between mothers reading newspaper/magazine at least once a week and EIB in Papua New Guinea, which is similar to a previous study in Ethiopia on the association between exposure to mass media and EIB [60]. Specifically, it was noted that women who read newspaper at least once a week had lower odds of EIB. This finding, however, contradicts findings from a study conducted in Ghana by Duodu et al. [32] which reported no statistical significance between watching television, listening to radio, and EIB. The association between exposure to newspaper and EIB foregrounds the need for further studies to unravel the nuances since newspaper reading is reducing due to the growth of the internet and the provisions of digital information sources [61].

Again, as shown in previous studies in Ghana [32], Kenya [48], Nepal [49], Nigeria [23], and Saudi Arabia [24], we observed that region of residence is associated with EIB, with women from the Islands region having lower odds of EIB compared to those in Southern region. The Southern region of Papua New Guinea is predominantly developed. It contains the city of Port Moresby, which is the capital of the country and the center for major businesses. On the other hand, the Islands region is less developed and contains least densely populated areas in the country [62–64]. A study by Woldeamanuel [65] reported that mothers in urban or developed areas in Ethiopia are 29% more likely to practice EIB than mothers in the less developed areas. Similar findings have been reported by Bbaale [66] in Uganda, Yahya and Adebayo [67] in Nigeria, and Victor et al. [68] in Tanzania. The regional disparities of EIB in Papua New Guinea could be as a result of the disparities in the allocation of developmental projects and health amenities across the regions in the country. For instance, residents in the southern region have more and easy access to maternal health information, education, and services as compared to their counterparts in the Islands region, which could lead to high EIB [64]. This finding highlights the need for policies and programs aimed at improving EIB to pay attention to cross-regional variations in EIB. Specifically, such policies need to focus attention on women from deprived regions of Papua New Guinea.

### Strength and limitations

The major strength of this study is the use of a large nationally representative sample which adheres to rigorous methodology and the use of validated instruments. This makes the findings generalizable to childbearing women in Papua New Guinea. Nonetheless, there is the possibility of recall-biased responses. With regard to the breastfeeding initiation, time after birth might introduce error in reporting by mothers. However, since giving birth is one of the memorable events for mothers, we assume such error would be minimal. The study employed cross-sectional design and, therefore, causality of the findings cannot be claimed. We also acknowledge that the determinants discussed in the paper are not new in the global context, they are rather offering support for earlier studies.

## Conclusion

The prevalence of EIB in Papua New Guinea was relatively high (60%). The factors associated with EIB are age of the women, region of residence, mode of delivery, place of delivery, practice of skin-to-skin contact with the baby. To scale up EIB in Papua New Guinea, these factors ought to be considered in the implementation of policies and strengthening of existing ones. There is the need for health professionals to educate mothers on the importance of EIB. Further studies could explore place of delivery, type of delivery and EIB.

## Data Availability

The dataset can be accessed via this link: https://dhsprogram.com/data/dataset/Papua-New-Guinea_Standard-DHS_2017.cfm?flag=0

## Abbreviations

EIB: Early Initiation of Breastfeeding
COR: Crude Odds Ratio;
AOR: Adjusted Odds Ratio
CI: Confidence Interval
PDHS: Papua New Guinea Demographic and Health Survey (PDHS)
VIF: Variance Inflation Factor
